# Supplementary material for: MetaRibo-Seq measures translation in microbiomes
Source: Nat Commun. 2020 Jun 29;11:3268. doi: 10.1038/s41467-020-17081-z (PMC7324362; doi:10.1038/s41467-020-17081-z)
Supplement: Supplementary file 10 — Supplementary Data 7 [file 41467_2020_17081_MOESM10_ESM.zip › File2/Confidence_High_Taxonomy/78146_out.krona.html]

Javascript must be enabled to view this page.

members
magnitude
magnitudeUnassigned
count
unassigned
taxon
rank

78146\_out

2

2
superkingdom
2

2
phylum
976

class
2
200643

171549
2
order

1853231
family
2

1
283168
2
genus

SRS144297\_contig\_number\_5877

1
species

SRS018836\_contig\_number\_21683
28118
